# Porterville Alternate Care Site Provision of Oxygen at COVID-19 Pandemic Peak

**DOI:** 10.1017/dmp.2022.172

**Published:** 2022-08-05

**Authors:** Lance Montauk

**Affiliations:** Division of General Internal Medicine, University of California, San Francisco (UCSF), CA, USA

**Keywords:** COVID-19, Oxygen, Logistics, California, Alternate Care Site

## Abstract

During the COVID-19 pandemic peak, the author deployed twice to an emergency Alternate Care Site in Porterville, California. The provision of oxygen to patients there, as seen from a physician’s perspective, does not fully support the description in a recently published article of how the State of California approached oxygen logistics during the COVID-19 surge. To inform future planning, an adequate logistical assessment must include not only approaches for solving technical resource challenges, but also reliable numbers regarding end-user resource utilization, and non-utilization, as well as program costs, benefits, and unintended consequences.

Disasters are unique. In a specific disaster scenario, the provision of matériel such as oxygen and pharmaceuticals, as well as other specific indicated services, can be challenging. Supplies may be allocated to both established medical centers and to temporary locations created to address the exceptional conditions. Successful execution of such programs requires logistical efforts utilizing physical resources, technical skills, and effort. However, even a flawlessly executed program can fail to yield the expected results, prove inordinately expensive, or even aggravate threats to public health. An after-the-fact objective assessment of a program, including its impacts both positive and negative, as well as its cost, should inform future planning.

California’s state-wide disaster approach to oxygen and ventilator logistics is well-described in Devereaux, Backer, *et al.’s* article *Oxygen and Ventilator Logistics during California’s COVID-19 Surge- When Oxygen becomes a Scarce Resource*.^1^ Given that the provision of oxygen to COVID-19 patients was a major rate-limiting step in patient hospital discharge, the State of California established Alternate Care Sites (ACSs) to provide care for those stable enough for typical hospital medical-ward level care, thus freeing up in-hospital beds for higher-acuity cases.

This brief report reviews the provision of care, centered upon patient oxygenation, during the COVID-19 pandemic program, as seen on-the-ground during operation of the California Office of Emergency Services’ (CalOES) temporary Alternate Care Site at Porterville (PACS) in California’s Central Valley. The report’s perspective is that of a California Medical Assistance Team (CAL-MAT) volunteer physician, who deployed twice to this location, during the peak of the pandemic (Figure 1).

**Figure 1.**
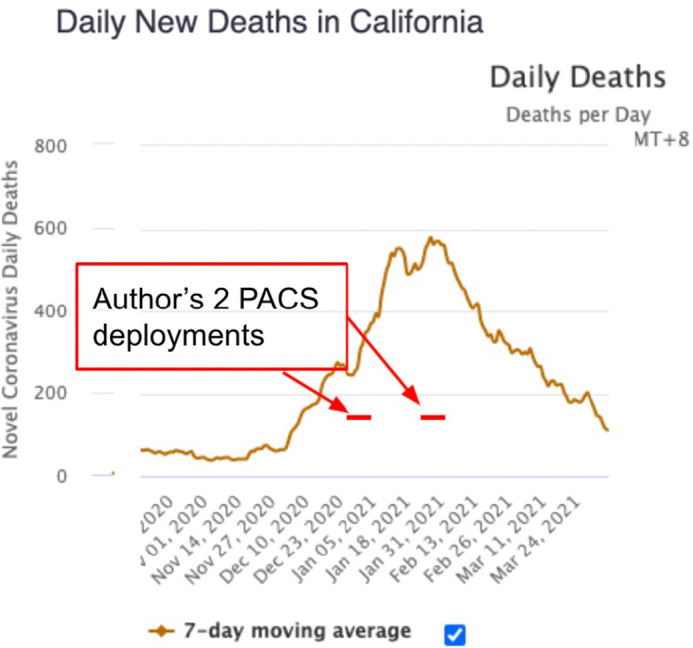
Daily deaths in California during author deployments.

## Patient census at facilities

**Figure 2.**
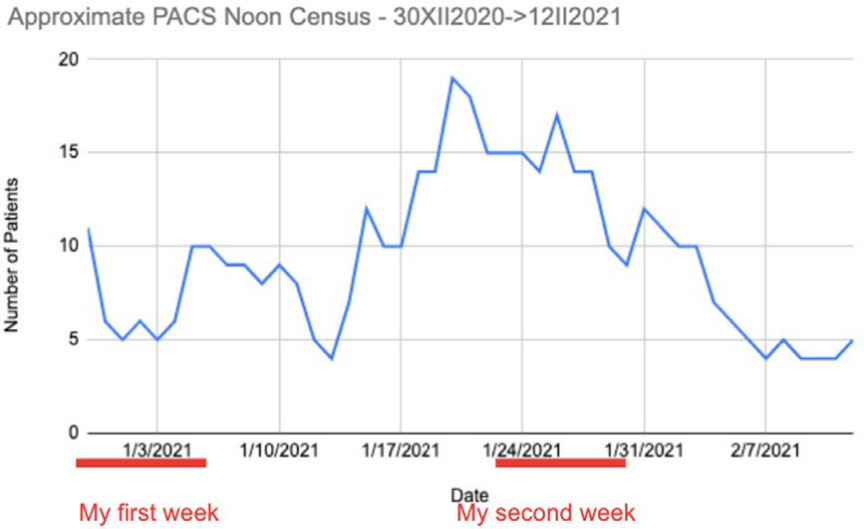
Daily patient census at PACS during pandemic peak.

**Figure 3.**
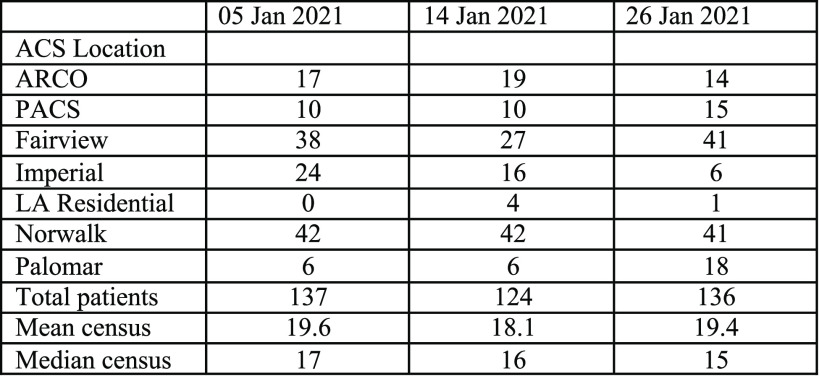
Number of patients at seven ACS sites during pandemic peak.

The graph below **(** Figure 2) shows that the daily census, at PACS never exceeded 19, and the typical noon census over 5 weeks, during the very peak of the epidemic, was approximately 10 patients.^a^ The total number of patients at all 7 ACS sites combined (data from 3 CalOES press reports) during the pandemic’s peak, show total statewide census never reached 140^b^ (Figure 3).^2–4^ The average census of all 7 sites was approximately 20 patients/site at the peak of the epidemic; over 50% the patients were at just 2 of the sites.

## Utilization of personnel

**Figure 4.**
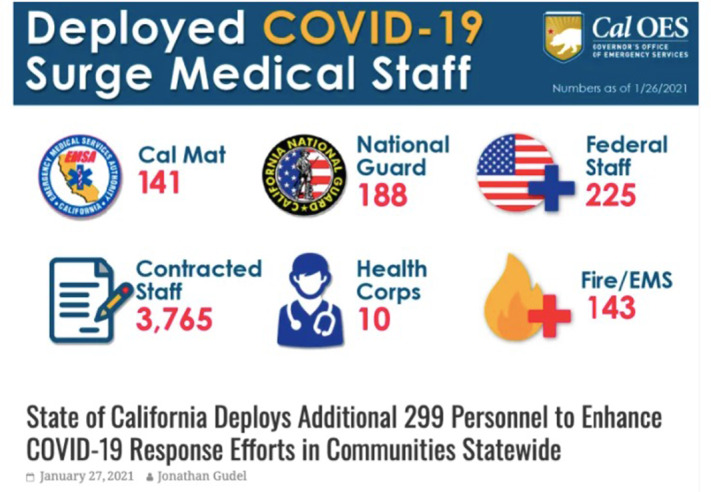
CalOES press release showing deployed staff at all 7 ACS sites, January 27, 2021.

Provision of care at the ACS sites required adequate staff. At its peak, CalOES mobilized 4472 individuals to provide care for fewer than 140 individuals, a ratio of 31 mobilized personnel for each individual in-patient during pandemic peak, and an even higher ratio at non-peak times (Figure 4).^c^

## Equipment never utilized

**Figure 5.**
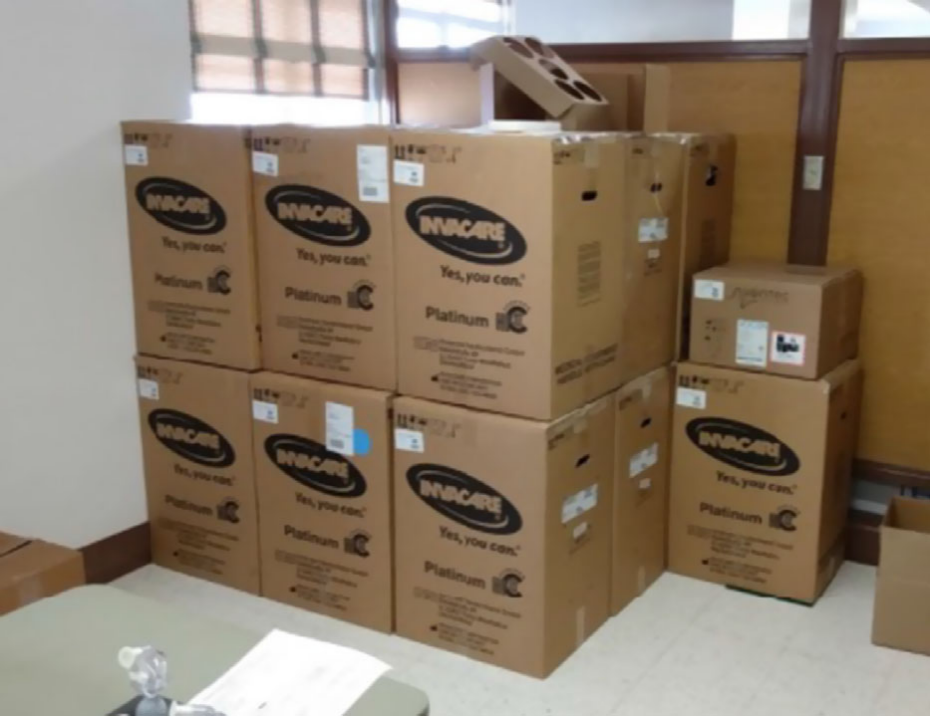
Nineteen unopened oxygen concentrator boxes at PACS, December 30, 2020.^d^

**Figure 6.**
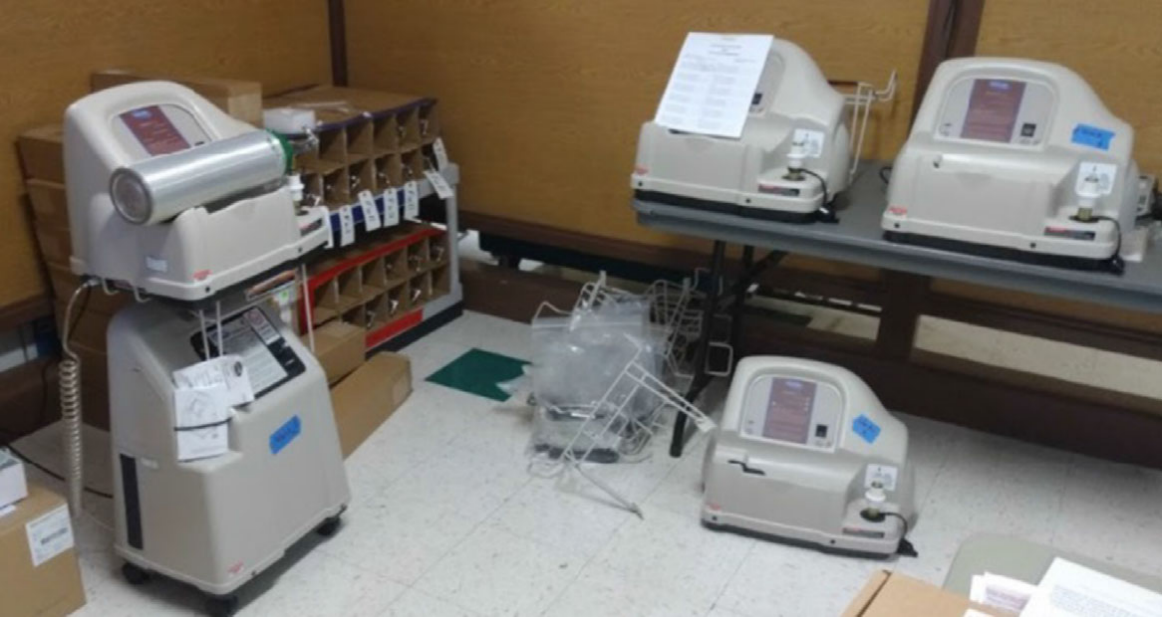
One utilized and three non-utilized oxygen tank fillers, along with dozens of oxygen bottles, most still in unopened boxes.

Devereaux *et al.* do not discuss how much oxygen equipment was acquired but never actually utilized. Almost 50% of the oxygen concentrators and tanks at the PACS site were never taken out of their new boxes/packages or put in service. Presumably the same held true for some other sites as well (Figures 5, 6, & 7 below of unused equipment [oxygen concentrators, tanks, etc.] during the pandemic peak).

**Figure 7.**
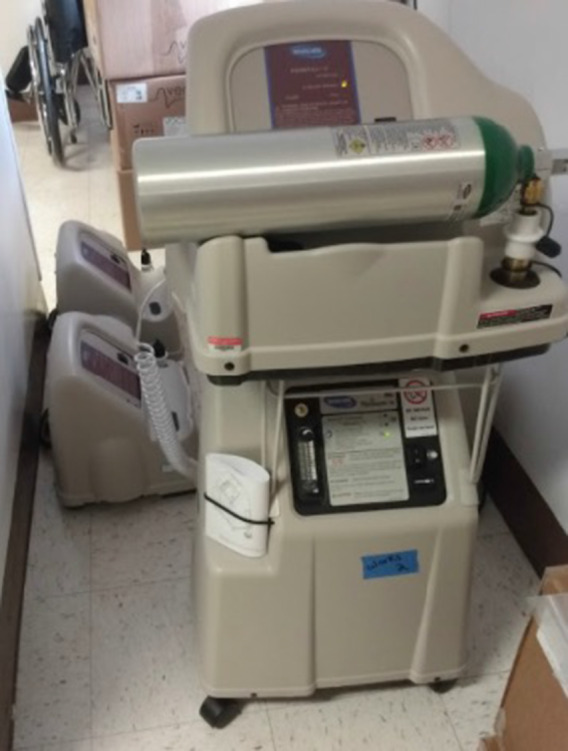
Three more oxygen tank fillers, one of which was utilized.

## Equipment availability

### Oxygen concentrators

**Figure 8.**
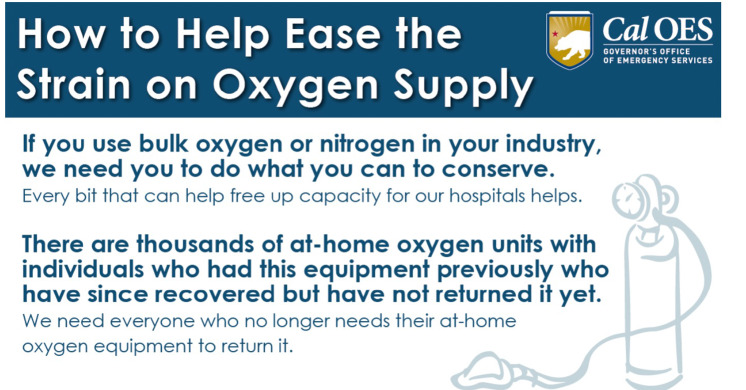
Press release from CalOES, January 25, 2021.

According to Devereaux *et al.*, approximately 500 oxygen concentrators were acquired by CalOES. Of these, given patient census numbers and the utilization pattern at PACS described above, perhaps 50% (250) were never utilized. While the removal of these 250 concentrator units from a market as large as California arguably had a minimal effect, the acute shortage of home oxygen equipment prompted a press release by CalOES urging individuals with oxygen equipment no longer in use at home to ‘return’ it (Figure 8).

**Figure 9.**
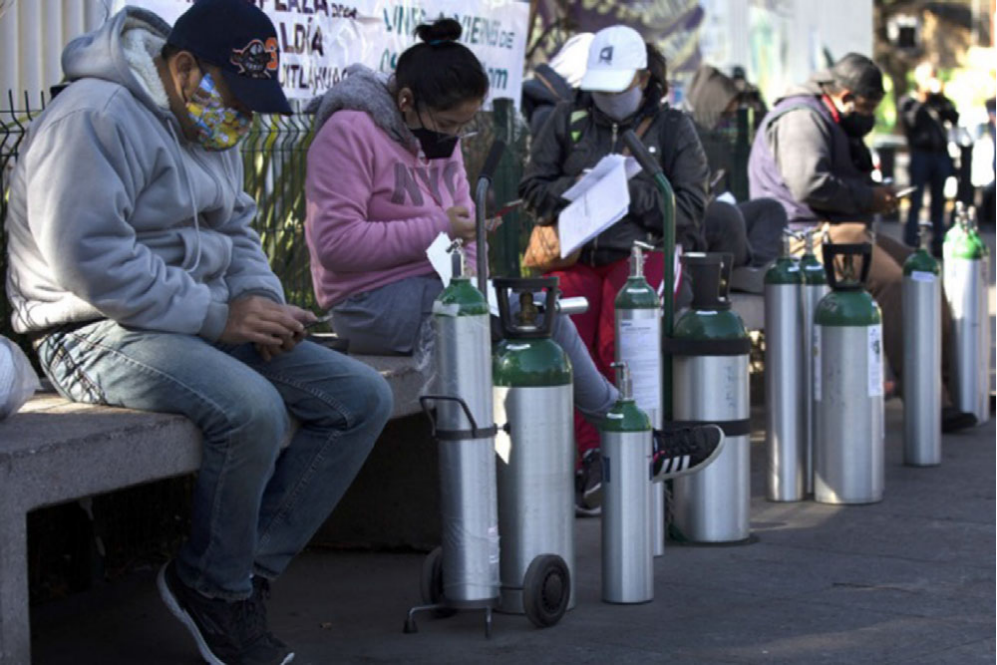
A Mexico City Queue for Oxygen Bottles (AP, Marco Ugarte, January, 2021).

**Figure 10.**
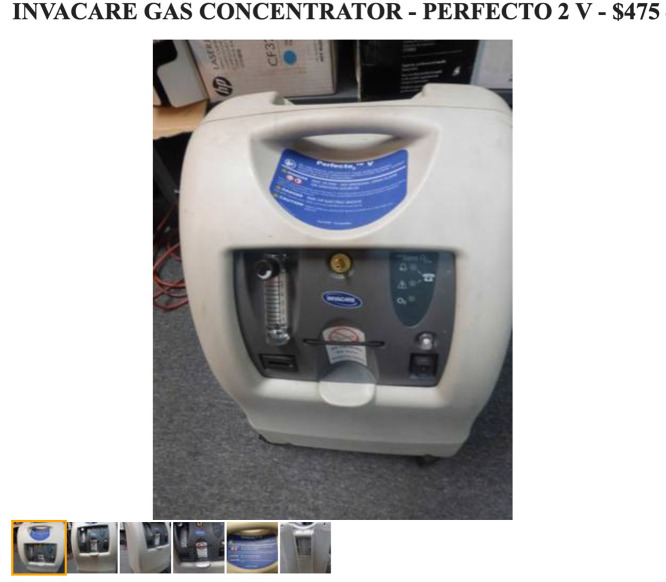
A typical Craigslist listing of an oxygen concentrator for sale, February 2022.

Unfortunately, oxygen shortage impacts did not stop at either the state or national border. In late January 2021, the then-ongoing, pandemic-triggered, oxygen shortage in Mexico caused Mexican expats in Northern California to snap up used oxygen concentrators on secondary markets such as Craigslist, to personally transport them 3000 km to Mexico City (Figures 9 & 10; author’s personal data).

### Ventilators

Given their status as general medical ward care-providers only, ACSs such as PACS did not use ventilators for patient care. Thus, the author can provide no direct observational data regarding utilization of the 15000 ventilators which CalOES purchased from 10 different manufacturers. While Deveraux *et al.* report that acquisition, they provide no information regarding how many of those units were distributed to hospital end-users, nor how many of that number were put into service, and how many patients used them, etc. Without such numbers, it is impossible to assess the utility of that logistical aspect of the emergency provision of oxygen even casually.

## Conclusion

Description of the logistical hurdles to providing oxygen to California’s healthcare system during the COVID-19 pandemic is a worthwhile undertaking, but without considering the efficiency of the efforts nor their eventual positive and negative impacts, few conclusions are possible.

Complete logistical description must quantify how much material was lost, stolen, or never utilized, how much the effort cost, what were the measurable or estimated benefits, and what were the negative side-effects, intended, or otherwise.

Without such a complete perspective, future proposals to provide oxygen supplies to patients in pre-existing or stand-up emergency facilities, such as the CalOES Alternate Care Sites, will be evaluated in the dark. Improved preparation for future events entails an honest and complete appraisal of prior efforts.

## Footnotes

a Graph displays PACS noon census numbers obtained on a daily basis by the author, with data taken from the PACS dashboard; these numbers tally closely with those from CalOES press releases.

b Ninety days earlier, in early April, total census for all 7 ACS sites combined was 27 patients, i.e., an average of 4 patients per site. Devereaux *et al.*'s assertion that the census at individual locations ranged from 20-50 patients appears exaggerated when compared with *peak* numbers (their article refers to 5 ACSs although it appears 7 were operational). Outside of peak periods the numbers asserted appear equally or more dubious.

c The author cut short the first deployment (originally scheduled for two weeks) when the ratio of doctors to patients at PACS approached 1:1.

d At PACS census peak, 15 concentrators remained un-utilized for patient care, i.e., slightly over half were put into service.
